# The global diet quality score (GDQS) of foods consumed by Nigerian adults

**DOI:** 10.1186/s41043-025-00764-y

**Published:** 2025-03-08

**Authors:** Galya Bigman, Sally N. Adebamowo, Nutrition Epidemiology Research of Nigeria (NERON) Group, Clement A. Adebamowo

**Affiliations:** 1 Department of Epidemiology and Public Health, University of Maryland School of Medicine, Baltimore, MD USA; 2 Greenebaum Comprehensive Cancer Center, University of Maryland School of Medicine, Baltimore, MD USA; 3 Department of Research, Center for Bioethics and Research, Ibadan, Nigeria

**Keywords:** Global diet quality score, Nigeria, Seasons, Healthy foods, Unhealthy foods

## Abstract

**Background:**

Poor diet quality is a significant and modifiable risk factor associated with numerous non-communicable diseases. Despite its critical importance, there is a paucity of comprehensive data concerning diet quality in Nigeria. In this study, we evaluated the healthiness of food intake among Nigerian adults to identify the factors associated with them and seasonal variations in food consumption patterns.

**Methods:**

We used a validated semi-quantitative Food Frequency Questionnaire (FFQ). to collect dietary data from adults in Ibadan, Nigeria, on four occasions over two years. We assessed food intake healthiness using the Global Diet Quality Score (GDQS), which ranges from 0 to 49 based on 25 food groups, and its sub-metrics GDQS + (0–32, 16 food groups) and GDQS − (0–17, 9 food groups). We used Generalized Linear Models (GLMs) to examine the relationships between GDQS, demographic factors, and participants’ dietary habits.

**Results:**

There were 205 participants (110 women, 95 men) with mean(SD) age of 45.0(13.4) years and mean(SD) GDQS of 29.0(4.0). Some 91.7% of the participants had a GDQS ≥ 23, signifying a low risk of poor diet quality. The multivariable analysis showed that the GDQS of those who frequently consumed home-cooked meal was higher than those who did not by 2.04 (95%CI: 0.11 to 4.07). The GDQS + of men was higher than women by 1.64 (95%CI: 0.11 to 3.03). The GDQS − of men was 0.88 lower than that of women (95%CI: − 1.53 to − 0.24), while younger participants (< 40 years) had lower GDQS − compared to older (60 + years) participants (2.51, 95%CI: − 1.58 to − 3.43) indicating a higher intake of unhealthy foods by these groups. Most participants (~ 95%) reported low intake of cruciferous vegetable.

**Conclusions:**

Despite the overall healthiness of foods consumed by Nigerian adults and low risk of poor diet quality across seasons, men and younger adults tend to consume more unhealthy foods and fewer home cooked meals. Both genders, irrespective of age, had low intakes of cruciferous vegetables. These findings highlight opportunities for targeted interventions to improve the overall healthiness of dietary intakes among Nigerian adults.

## Background

The global incidence of diet-related non-communicable diseases (NCDs), including obesity, hypertension, and diabetes, is rising steadily. This trend is also evident in Nigeria, where the prevalence of overweight and obesity is 26.3% and 10.9% among men, and 28.3% and 23.0% among women [1–3]. The age-standardized prevalence of hypertension in Nigeria is estimated at 28.9% (95% CI: 25.1–32.8), while the prevalence of diabetes has more than doubled over the past two decades, increasing from 2.0% (95% CI: 1.9%–2.1%) in 1990 to 5.7% (95% CI: 5.5%–5.8%) in 2015 [1–3]. A key driver of this growing NCD burden is the changing dietary patterns, which have significantly altered the healthiness of foods consumed by Nigerians [4–8].

Dietary intakes in Nigeria are influenced by the country’ s geographical, social, and cultural diversity, as well as the seasonal availability of foods [5–11]. In the southern region, starchy roots and tubers, such as yams and cassava, along with their derivatives like garri, fufu, and lafun, serve as staple foods. In contrast, the northern region predominantly relies on grains such as sorghum and millet. In both regions, these staple foods are typically accompanied by oil-based soups enriched with tomatoes and vegetables [5]. More recently, there has been a notable increase in the consumption of rice and wheat-based products [6]. The distinct climatic seasons, marked by dry and rainy periods, further influence the availability of locally sourced foods, which in turn impacts dietary choices across the country [5–12].

While traditional Nigerian diets are generally considered healthful, there has been a notable shift toward less healthy dietary patterns, driven by urbanization, technological advancements, social media, and improved transportation. These factors have contributed to an evolving dietary landscape, marked by the introduction of new food choices that are altering traditional eating habits [8]. This transition is characterized by a significant increase in the consumption of processed, calorie-dense foods, including refined grains (such as white bread), pastries, sugary beverages, and deep-fried foods [5–9]. Although traditional Nigerian dishes are still offered in the growing number of fast-casual dining and takeaway restaurants, these establishments are playing a key role in the ongoing nutrition transition [4]. As a result, a complex array of factors is shaping contemporary dietary patterns in Nigeria.

While significant efforts have been made to assess the dietary quality of Nigerian adults, there remains limited research on the variability in the healthiness of overall food consumption [13, 16]. Although some studies have explored dietary habits and their effects on health in Nigeria and other African countries, a critical gap exists in our understanding of diet quality. This gap is largely due to the lack of validated dietary assessment tools tailored to capture the unique aspects of African diets and culinary traditions. Furthermore, the absence of regularly updated and consistent food composition databases exacerbates this challenge [11, 13, 14]. A new approach for assessing the quality of dietary intakes across different populations is the Global Diet Quality Score (GDQS) [15, 16]. Designed to identify associations between diet quality, nutrient adequacy, and non-communicable disease (NCD) risk, the GDQS is entirely food-based, thus bypassing the need for a food composition database—a resource often insufficiently maintained or absent in low- and middle-income countries (LMICs) [11]. Unlike traditional dietary metrics, the GDQS incorporates a wide range of both healthy and unhealthy food groups and includes consumption quantity in its scoring framework. This enables a more comprehensive evaluation of dietary patterns, facilitates global comparisons of food consumption healthiness, and provides valuable insights into the dietary habits of the study population, while also comparing them with those of other populations globally [15].

This study aimed to assess the overall healthfulness of common food choices among Nigerian adults. Additionally, we sought to identify the factors influencing healthy food intake and to assess whether these factors varied across different seasons in the country. To achieve these objectives, we employed a cross-sectional study design, utilizing surveys and dietary assessments to gather data on food choices and associated influencing factors.

## Methods

### Study sample and setting

We employed a purposive sampling strategy to recruit 220 adult participants aged 18 years and older from Ibadan, southwestern Nigeria, to validate our FFQ as previously described [14]. Our sample size was determined by considering a confidence interval of 95%, a study power of 80%, a minimum expected correlation coefficient of 0.25, an estimated attrition rate of 50%, and anticipated dropout rate of 10% [17]. We meticulously designed the sampling to ensure a diverse and representative study sample by considering variables such as age, gender, occupation, and ethnicity/tribe to capture a broad range of food consumption patterns. Our objective was to achieve proportional representation across the three major Nigerian tribes—Yoruba, Hausa-Fulani, and Igbo—while reflecting the socio-economic and age demographics of the Oyo State region, where Ibadan is situated [18]. This approach enhanced our ability to encompass a wide range of perspectives and characteristics within the target population. Participants gave informed consent prior to their enrollment in the study. The study was approved by the Nigerian National Health Research Ethics Committee (NHREC) Approval Number NHREC/01/01/2007–05/06/2024E; 05/06/2018, and the Institutional Review Board (IRB) of the University of Maryland Number HP-00082169-18122 GCCCC AFBRECANE; 09/10/2018.

### Study sample characteristics

We gathered data on age in years, years of education completed (< 11 years of school, 12 years of school, post-secondary school and university), occupation (Unemployed, Self-Employed, Skilled Manual, Professional/Executive), Tribe (Yoruba, Igbo, Hausa/Fulani, Other), and dietary habits such as how often home-cooked meals were consumed and the most and least common locations for food consumption. Eligible participants were individuals aged over 18 years who could complete the FFQ in either English or their preferred predominant indigenous language. Exclusion criteria included pregnant and lactating women, as well as individuals with major medical conditions. The selection criteria were strategically designed to ensure a well-balanced participant group.

### Dietary intake data

We used a validated semi-quantitative Food Frequency Questionnaire (FFQ), with a reproducibility test showing a mean ± SD of 0.39 ± 0.14 and a validity of 0.27 ± 0.16 for the intake of the most common Nigerian food items, to measure participants’ food intake at baseline and every six months over four assessments between November 2018 and October 2020. Baseline and 12-month measurements were conducted during the dry season (November to March), while the 6-month and 18-month measurements occurred during the rainy season (April to October). Briefly, the FFQ is complemented with a Food Picture Book (FPB) featuring typical Nigerian foods and their corresponding standardized portion sizes. Further details on the Nigerian FFQ and FPB can be found elsewhere [14]. The FFQ includes about 200 food items where frequencies of their consumption were reported on a monthly, weekly, and daily basis ranging from “Never or less than once per month” to “6 or more times per day.” Participants were asked to choose the option that most accurately represents their typical or average consumption of the listed food item during the past year. Each reported food items in number of portions were then converted to grams per day as follows: frequency of intake × conversion factor for daily intake × total number of portions × portion weight. Conversion factors were derived from intake frequency, adjusted to represent daily intake. For instance, if a certain food was reported to be consumed 2–4 times a week, we multiplied the frequency by 3/7 to convert it to daily intake. All FFQs and FPB were administered to participants and completed face-to-face by the same trained personnel, typically in the participants’ homes. No data were collected during holidays, festivals, or weekends. All data were doubly entered into the Research Electronic Data Capture (REDCap) database [19, 20].

### The global diet quality score (GDQS)

We employed the GDQS approach to evaluate the healthiness of food consumption in our study population. GDQS analyze diet data at the food group level to gain insights into the types of foods consumed at the population level. After we calculated the daily food intake in grams for each food reported at the FFQs, we categorized them into their respective GDQS food groups as shown in Table 1. Mixed dishes reported in the FFQ, such as soups like egusi and bitter leaf, were disaggregated into individual foods using standard recipes [21]. For simple dishes with up to three ingredients, such as white rice, breakfast cereal, and ‘ swallows,’ we estimated the weights of each ingredient in the prepared foods. For instance, the weight of one portion of prepared oats with water/milk was 456 g; this was multiplied by one-fifth to estimate the weight of the oats alone and the value obtained was used to calculate the white grain intake in this food.

**Table 1:** GDQS and GDQS sub-metrics (GDQS + , GDQS −) food groups and scoring and all the applicable food items collected from the FFQ

		Food group	Scoring ranges (g/day)	Respective point values	Applicable food items from the FFQ
Included in the GDQS	Included in the GDQS+	Citrus fruits	< 24, 24–69, > 69	0,1,2	Orange; Tangerine; Lemon; Lime
		Deep orange fruits	< 25, 28–123, > 123	0,1,2	Mango; Pawpaw; Apricot
		Other fruits	< 27, 27–107, > 107	0,1,2	Banana; Apple; Guava; Plum; Peach; African Pear (IG: Ube); Avocado; Passion fruit; Tamarind (HA: Tsamiya); African cherry (YR: Agbalumo); Pineapple; Grapes; Watermelon; Jackfruit (Bread fruit; Sweet melon; Berries; Baobab (HA: Kuka fruit); Fruit salad; Plantain
		Dark green leafy vegetables	< 13, 13–37, > 37	0,2,4	Waterleaf, (Vegetable soup, Afang, Utazi, Edi ka ikong, Uziza); Pumpkin leaf (Egusi soups); Bitter leaf (Bitter leaf soup, banga soup); Amaranthus leaves (HA: Ayoyo, YR: Ewedu))
		Cruciferous vegetables	< 13, 13–36, > 36	0, 0.25, 0.5	Coleslaw
		Deep orange vegetables	< 9, 9–45, > 45	0, 0.25, 0.5	Carrot; Pumpkin, yellow and orange squash
		Other vegetables	< 23, 23–114, > 114	0, 0.25, 0.5	Cucumber; Garden egg; Vegetable salad; Okra
		Legumes	< 9, 9–42, > 42	0,2,4	Bean alone; Beans porridge; Bean cake (YR: Akara); Corn with beans (YR: Adalu); Bean pudding (YR: Moinmoin); Soya drink; Bean Soup (Gbegiri)
		Deep orange tubers	< 12, 12–63, > 63	0, 0.25, 0.5	Sweet potato – Boiled/Fried; Potato porridge (sweet potato)
		Nuts and seeds	< 7, 7–13, > 13	0,2,4	Groundnut (Cooked/Roasted); Cashew nut; Tiger nut (YR: Ofio, HA: Aya, IB: Akiausa); Walnut (YR: Asala); Kwuli-kwuli; Peanut butter
		Whole grains	< 8, 8–13, > 13	0,1,2	Oats (i.e., Quaker Oats); High fiber cereals i.e., Bran; Brown rice; Corn (Roasted/Boiled); Corn and beans (YR: Adalu); Tuwon masara (swallows); Pap from corn (YR: Akamu, Ogi); Millet meal (Pap); Tuwon dawa (guinea corn) (swallows); Tuwon gero (millet); Popcorn
		Liquid oils	< 2, 2–7.5, > 7.5	0,1,2	Palm oil
		Fish and shellfish	< 14, 14–71, > 71	0,1,2	Snail; Shrimp and Prawns; Fish Sea-Water (boiled/fried); Fish Fresh-Water/River (boiled/fried); Dried/Smoked fish; Sardines
		Poultry & game meat	< 16, 16–44, > 44	0,1,2	Chicken (with/without skin)—Broiled, fried or grilled; Turkey; Guinea fowl
		Low-fat dairy	< 33, 33–132, > 132	0,1,2	Fresh milk; Chocolate drink (Milo, Bournvita etc.) with milk; Custard
		Eggs	< 6, 6–32, > 32	0,1,2	Chicken egg (Boiled/ Fried)
	Included in the GDQS −	High-fat dairy	< 35, 35–142, > 140–734, > 734	0, 1, 2, 0	Evaporated liquid milk; Powdered milk; Yoghurt (plain/sweet); Cream cheese
		Red meat	< 9, 9–46, > 46	0,1,0	Beef (boiled/fried); Goat Meat (boiled/fried); Pork (Pig meat)(boiled/fried); Lamb/Mutton)(boiled/fried); liver (boiled/fried); Offal/Tripe (YR: orisirisi)
		Processed meat	< 9, 9–30, > 30	2,1,0	Bushmeat; Bacon; Processed cow skin (YR: Ponmo, Bokoto, Cow-Leg); Canned meats (Bully beef/Corned beef); Meat minced; Suya
		Refined grains and baked goods	< 7, 7–33, > 33	2,1,0	Spaghetti; Noodles (e.g. Indomie); Macaroni; Rice (White, Jollof, Fried, Ofada, Coconut); Ground rice (Rice flour) swallow; Wheat flour swallow; Tuwon shinkafa; Bread (sliced; flat); Meat Pie (Meat samosa), e.g. Gala; Bread rolls; Pancakes; Vegetable samosa; Doughnut, Fried dough, Buns, Puffpuff; Semolina; Breakfast cereal e.g. Cornflakes, Rice Krispies
		Sweets and ice cream	< 13, 13–37, > 37	2,1,0	Ice cream; Cake, tarts, scones, muffins; Chocolate bar; Sugar added to foods (include in tea & coffee); Honey; Jam; Marmalade; Sugarcane; Raisins (Cake fruit); Dates (HA: Dabbino)
		Sugar-sweetened beverages	< 57, 57–180, > 180	2,1,0	Soda—regular (Coke, Fanta, etc.); Soda – Diet; Chocolate drink (Milo, Bournvita etc.) without milk
		Juice	< 36, 36–144, > 144	2,1,0	Orange or other fruit juices (sweetened/ unsweetened) Fruit squash, concentrate – mixed with water (sweetened/with artificial sweetener)
		White roots and tubers	< 27, 27–107, > 107	2,1,0	Irish potato (boiled/fired); Boiled Cassava; Potato porridge (Irish potato); Yam porridge; Yam (boiled/fired); Traditional Pounded Yam; Pounded Yam from Flour; Garri; Eba (Swallows); Cassava flour swallow (YR: lafun); Cocoyam (Boiled/Chips); Amala (swallows); Fufu or Akpu (swallows)
		Purchased deep fried foods	< 9, 9–45, > 45	2,1,0	Potato or Corn chips or crisps; Cocoyam chips; Doughnut, Fried dough, Buns, Puffpuff; Plantain chips (YR: Igbekere/Ipekere); Fried Sliced Plantain (YR: Dodo); Fried Yam

HA, Hausa/Fulani; IB, Igbo; YR, Yoruba; GDQS; global diet quality score; GDQS + , healthy food groups; GDQS − , unhealthy food groups

The GDQS consists of 25 food groups, categorized based on their impact on diet quality, with each food item assigned to the appropriate group. Purchased deep-fried items were "double-counted," in deep-fried foods group and another group based on their characteristics. For example, fried yam (Dundu) was in deep-fried foods and in the white roots and tubers groups. We computed the overall daily intake of each GDQS food group in grams. They were then further categorized into GDQS + (healthy foods), which includes 16 food groups that positively contribute to the overall diet quality score. (Table 1), and GDQS − (unhealthy foods) comprising 7 food groups that have a negative impact on the overall diet quality score (Table 1). Additionally, 2 food groups (red meat and high-fat dairy) were classified as "unhealthy in excessive amounts," where optimal intake improves while excessive intake reduces the overall diet quality score.

Values corresponding to each GDQS food group were assigned according to daily intakes (Table 1). Specifically, each food in GDQS + were assigned points ranging from 0 to 4 for each level of intake, while foods in GDQS − were assigned 2, 1, and 0 points. This scoring system ensures that higher intakes of healthy foods and lower intakes of unhealthy foods resulted in higher GDQS scores. Red meat and high-fat dairy are assigned a score of 0 when they are consumed in either low or excessive amounts. Red meat is assigned a score of 1 when consumed in moderate amounts. High-fat dairy received scores of 0, 1, 2, and 0 across four levels of intake where 0 is assigned for low amounts intake and 0 for ‘ unhealthy in excessive amounts.’ The total possible score for the overall GDQS ranged from 0 to 49 while that of the GDQS + ranged from 0 to 32, and that of GDQS − ranged from 0 to 17. The overall GDQS and its sub-metrics (GDQS + and GDQS − ) offer insights into the balance between healthy and unhealthy foods, with GDQS helping to identify individuals at low risk (GDQS ≥ 23), moderate risk (23 > GDQS ≥ 15) and high risk (GDQS < 15) of poor diet quality. For additional information please refer to these references [12, 22].

### Statistical analysis

We used STATA 18.0 (STATA Corp LP) for data analyses and set statistical significance at *p*-value < 0.05. Dietary intakes were assessed and analyzed based on the FFQ administered at baseline using cross-sectional approach. When comparing the seasonality of GDQS and its sub-scores (GDQS + and GDQS − ), we used all four FFQs. Categorical variables are presented as frequencies and percentages, while continuous variables are presented as means and standard deviations. We reviewed outliers against the original questionnaire, and these were resolved where possible, and we excluded unresolved cases. Where participants gave data with inadequate time intervals between the questionnaires to ensure that we captured different seasons or years, the observations were excluded, resulting in a final sample of 205 out of the initial 220, which accounted for a response rate of 93%.

We employed chi-square analysis for categorical variables and t-tests for continuous variables. Based on the Shapiro–Wilk test, the GDQS data was not normally distributed (p < 0.001). Consequently, we employed the Kruskal–Wallis equality of population rank test to examine differences in GDQS and its sub-metrics (GDQS + , GDQS − ) across study characteristics and dietary habits. We utilized all four FFQs to assess differences in GDQS and its sub-metrics (GDQS + , GDQS − ) between men and women separately for the rainy and dry seasons, employing the Wilcoxon signed-rank test. In the final analysis, we employed Generalized Linear Model (GLM) utilizing the family (gaussian) option with the identity link function to investigate the relationship between GDQS and its sub-metrics as primary outcomes, while adjusting demographic characteristics, and dietary habits as independent variables in the multivariable models.

## Results

### Study characteristics

The characteristics of the study population, as determined by the baseline FFQ, are shown in Table 2. There were 205 participants, consisting of 110 (53.7%) women and 95 (46.3%) men. The age of participants ranged from 19 to 83 years with mean (SD) of 45.0(13.4) years. Women were slightly older with a mean (SD) age of 46.7(13.1) compared to 43.0(14.1) years for men (*p* = 0.01). The women were more likely to be self-employed (58.2%), whereas men were predominantly engaged in skilled manual jobs (62.1%) (*p* < 0.001). There were notable differences in dietary behaviors. Women were more likely to consume home-cooked meals, whereas approximately a quarter of the men seldom or occasionally indulged in home-cooked meals, with over a third predominantly consuming food outside the home, such as at workplaces or restaurants (*p* < 0.001).

**Table 2:** Baseline socio-demographic characteristics and dietary habits of study sample: total and by sex (N = 205)

		Totals N (%) N = 205	Women N (%) n = 110 (53.7)	Men N (%) n = 95 (46.3)	*p*-value*
*Socio-demographic characteristics*					
Age (years)					0.01
	19–40	74 (36.1)	31 (28.2)	43 (45.3)	
	41–50	57 (27.8)	30 (27.3)	27 (28.4)	
	51–60	47 (22.9)	34 (30.9)	13 (13.7)	
	60 +	27 (13.2)	15 (13.6)	12 (12.6)	
Tribe					0.57
	Yoruba	70 (34.1)	35 (31.8)	35 (36.8)	
	Igbo	45 (22.0)	24 (21.8)	21 (22.1)	
	Hausa/Fulani	79 (38.5)	43 (39.1)	36 (37.9)	
	Other	11 (5.4)	8 (7.3)	3 (3.2)	
Work					< 0.001
	Unemployed	21 (10.2)	7 (6.4)	14 (14.7)	
	Self-employed	80 (39.0)	64 (58.2)	16 (16.8)	
	Skilled manual	80 (39.0)	21 (19.1)	59 (62.1)	
	Professional/executive	24 (11.7)	18 (16.4)	6 (6.3)	
Education					0.21
	< 11 years of school	71 (34.6)	44 (40.0)	27 (28.4)	
	12 years of school	73 (35.6)	35 (31.8)	38 (40.0)	
	Post-secondary school University	61 (29.8)	31 (28.2)	30 (31.6)	
*Dietary habits*					
How frequently do you consume home-cooked meals?					< 0.001
	Rarely	3 (1.5)	0 (0.0)	3 (3.2)	
	Sometimes	26 (12.7)	5 (4.5)	21 (22.1)	
	Most of the time	118 (57.6)	65 (59.1)	53 (55.8)	
	All the time	58 (28.3)	40 (36.4)	18 (18.9)	
Where do you consume most of your meals?					< 0.001
	Home	159 (77.6)	98 (89.1)	61 (64.2)	
	Work	38 (18.5)	12 (10.9)	26 (27.4)	
	Restaurants/eatery	8 (3.9)	0 (0.0)	8 (8.4)	
Where do you consume least of your meals?					< 0.001
	Home	30 (14.6)	6 (5.5)	24 (25.3)	
	Work	47 (22.9)	17 (15.5)	30 (31.6)	
	Restaurants/eatery	128 (62.4)	87 (79.1)	41 (43.2)	

*P*-values less than 0.05 are considered statistically significant

^*^Chi-square analysis

### Food group intake

**Fig. 1:**
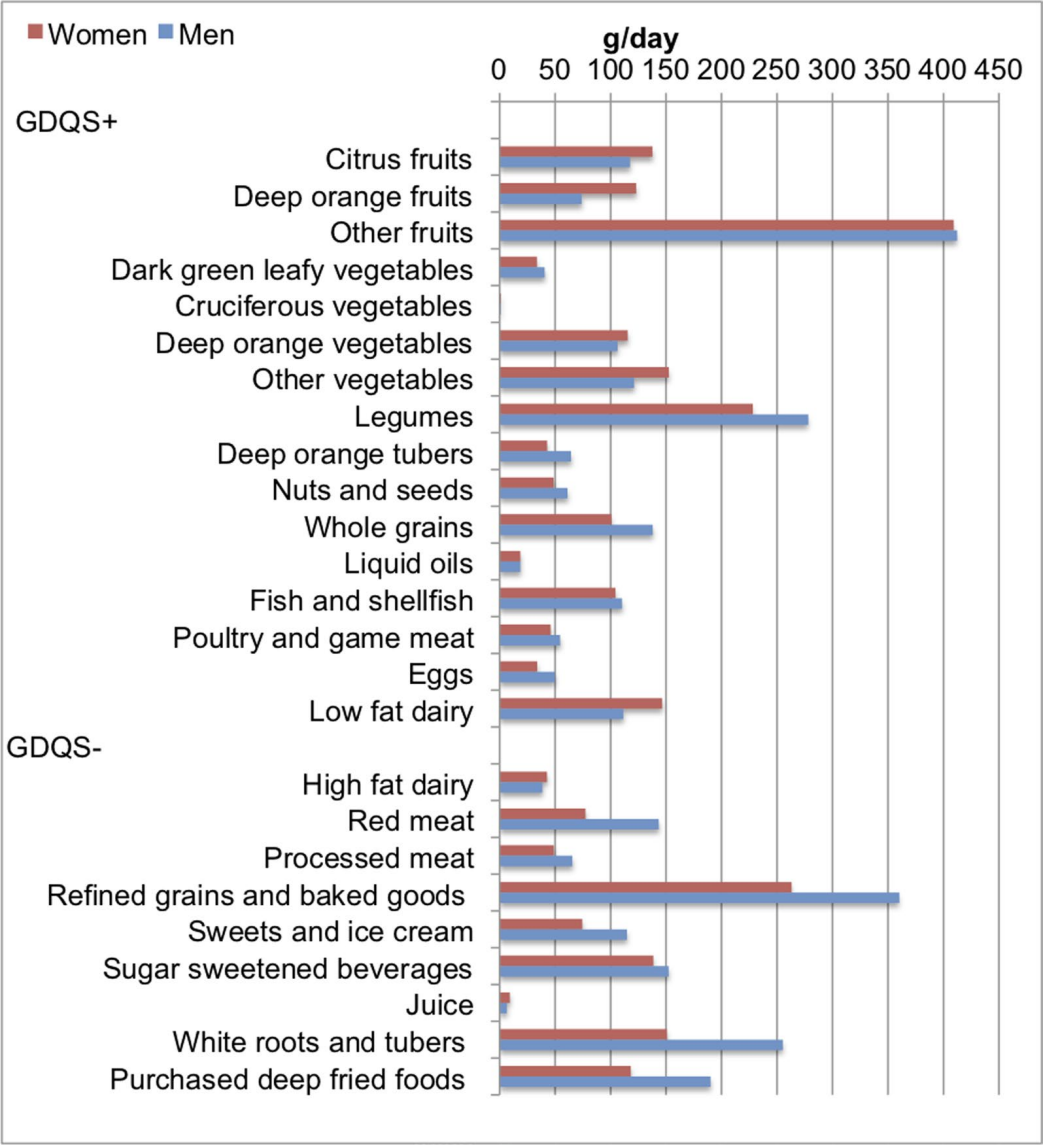
Gender differences in global diet quality score (GDQS) 25 food group intake (g = grams) baseline sample N = 205

Figure 1 shows the overall intake of GDQS food groups in grams per day, separately for men and women.

The mean (SD) amount of food groups in the diet of all the participants combined consisted 251.2 (163.8)g of legumes per day, 118.1(126.4)g of whole and refined grains per day, 639.0(511.6)g of fruits per day, 434.6(286.0)g of vegetables (including white or orange roots and tubers) per day and 152.4(160.0)g of deep-fried foods per day, while palm oil was the main source of liquid oil with mean(SD of 18.6(9.1)g consumed per day. The most common fruits consumed included banana, watermelon, cherry, pineapple, apple, and pear. Men consumed more refined grains such as white rice, bread, noodles, and breakfast cereal than women (360.2(181.5) vs. 263.0(182.4) g/day, *p* < 0.001), as well as more white roots and tubers such as yam and potatoes (255.1(157.8) vs. 150.7(113.1) g/day, *p* = < 0.001) compared to women. Women reported higher intake of deep orange fruits (123.0(213.6) vs. 73.8(113.2) g/day, *p* = 0.046), but had a lower consumption of dark green leafy vegetables (33.6(21.8) vs. 40.5(25.2) g/day, *p* = 0.04). The consumption of cruciferous vegetables among men and women was generally extremely low at less than 1.5 g per day and over 95% of the participants reported no consumption of cruciferous vegetables.

### GDQS and its sub-metrics

When combining all applicable food intake data to calculate the GDQS, we found that the mean (SD) overall GDQS for all participants was 29.0(4.0), with a median of 29.3 and an interquartile range of 27 to 32. There were no significant differences in mean (SD) GDQS when comparing men (29.1(3.9)) and women (29.0(4.1), p = 0.90), or across various study characteristics (Table 3). Overall, the majority of the participants (91.7%) reported a GDQS ≥ 23, indicating a low risk of poor diet quality, while the remaining 8.3% were at moderate risk (23 > GDQS ≥ 15). This proportion was slightly higher among men (93.7%) compared to women (90.0%) but this was not statistically significant (*p* = 0.34). Participants who reported consuming home-cooked meals all the time had a higher mean (SD) GDQS score of 30.0(3.6) compared to those who reported rarely/sometimes consuming home-cooked meals (28.3(4.6), *p* = 0.17), though this difference was not statistically significant.

**Table 3:** GDQS and its healthy (GDQS +) and unhealthy (GDQS −) sub-metrics by study characteristics. N = 205 baseline sample

			GDQS possible range: 0 to 49			GDQS + possible range: 0 to 32			GDQS − possible range: 0 to 17	
			Mean (SD)		*p*-value*	Mean (SD)		*p*-value*	Mean (SD)	*p*-value*
Total			29.0 (4.0)			23.0 (4.5)			5.8 (2.3)	
*Socio-demographic characteristics*										
Sex					0.9		0.04			< 0.001
	Men		29.1 (3.9)			23.8 (4.3)			5.3 (2.0)	
	Women		29.0 (4.1)			22.5 (4.6)			6.5 (2.5)	
Age (years)					0.26		0.01			< 0.001
	19–40		28.6 (4.1)			24.0 (4.7)			4.6 (1.8)	
	41–50		29.0 (4.6)			22.6 (5.0)			6.3 (2.6)	
	51–60		30.1 (3.0)			23.4 (3.4)			6.7 (2.2)	
	60 +		28.5 (3.8)			21.0 (3.6)			7.4 (2.5)	
Tribe					0.43		0.02			< 0.001
	Yoruba		29.2 (5.0)			22.6 (5.4)			6.6 (2.1)	
	Igbo		29.3 (3.2)			22.3 (3.8)			7.0 (2.0)	
	Hausa/Fulani		28.9 (3.4)			24.3 (3.8)			4.6 (2.1)	
	Other		28.5 (3.9)			21.2 (3.5)			7.3 (2.9)	
Work					0.12		0.12			0.03
	Unemployed		28.3 (3.9)			22.6 (3.9)			6.7 (2.9)	
	Self-employed		28.9 (3.5)			23.0 (4.3)			5.9 (2.7)	
	Skilled manual		29.1 (4.3)			23.6 (4.7)			5.5 (1.8)	
	Professional/executive		30.1 (4.6)			23.3 (4.6)			6.9 (2.0)	
Education					0.33		0.12			0.04
	< 11 years of school		28.6 (3.7)			22.7 (3.9)			6.0 (2.6)	
	11-12 years of school		29.5 (3.8)			24.0 (4.7)			5.5 (2.4)	
	Post school/university		29.0 (4.6)			22.6 (4.8)			6.4 (2.0)	
*Dietary habits*										
How frequently do you consume home-cooked meals?				0.17			0.2			0.01
	Rarely/sometimes		28.3 (4.6)			23.7 (5.2)			4.6 (2.1)	
	Most of the time		28.9 (3.9)			22.7 (4.4)			6.2 (2.3)	
	All the time		30.0 (3.6)			23.7 (4.0)			6.3 (2.6)	
Where do you consume most of your meals?				0.49			0.14			0.16
	Home		28.6 (3.7)			22.8 (4.5)			6.1 (2.4)	
	Work		29.0 (4.5)			24.0 (3.6)			5.5 (2.4)	
	Restaurants/eatery		29.2 (5.3)			24.7 (5.9)			4.8 (2.0)	
Where do you consume least of your meals?				0.29			0.1			0.09
	Home		29.2 (4.5)			24.1 (4.8)			5.1 (2.3)	
	Work		29.8 (3.7)			23.9 (4.1)			5.9 (2.2)	
	Restaurants/eatery		28.7 (4.0)			22.6 (4.5)			6.2 (2.4)	

GDQS, global diet quality score (25 food groups); GDQS + (16 healthy food groups- that should be consumed in high amounts) and GDQS − (9 unhealthy food groups- that should be consumed in low amounts)

*P*-values less than 0.05 are considered statistically significant

^*^Kruskal–Wallis equality of population rank test

However, when examining GDQS sub-metrics, we observed disparities between the sexes and across study characteristics, particularly in the consumption of unhealthy foods. The mean (SD) GDQS − was lower in men (5.3(2.0)) compared to women (6.5(2.5), *p* < 0.001), as well as in younger participants compared to older participants (4.6(1.8) vs. 7.4(2.5), *p* < 0.001). Significant GDQS − differences were also noted comparing those who consistently consumed home-cooked meals (6.3(2.5)) to those who rarely/sometimes did so (4.6(2.1), *p* = 0.01). Men consumed a higher proportion of nutritious food items (GDQS +), including eggs (47% vs. 41%, *p* = 0.02), poultry and game meat (38% vs. 25%, *p* = 0.01), and dark green leafy vegetables primarily in soups or cooked dishes (48% vs. 33%, *p* = 0.03). Women, on the other hand, reported higher intakes of other vegetables such as cucumber, garden egg, vegetable salad, and okra (52% vs. 36%, *p* = 0.048). Conversely, compared to women, men consumed a higher proportion of unhealthy foods (GDQS − ), particularly processed meats (63% vs. 42%, *p* = 0.01), red meats (90% vs. 76%, *p* = 0.01), sweets and ice cream (47% vs. 41%, *p* = 0.04), purchased deep-fried foods (79% vs. 65%, *p* = 0.04), and white roots and tubers (83% vs. 61%, *p* = 0.001).

### GDQS food groups by sex

**Fig. 2:**
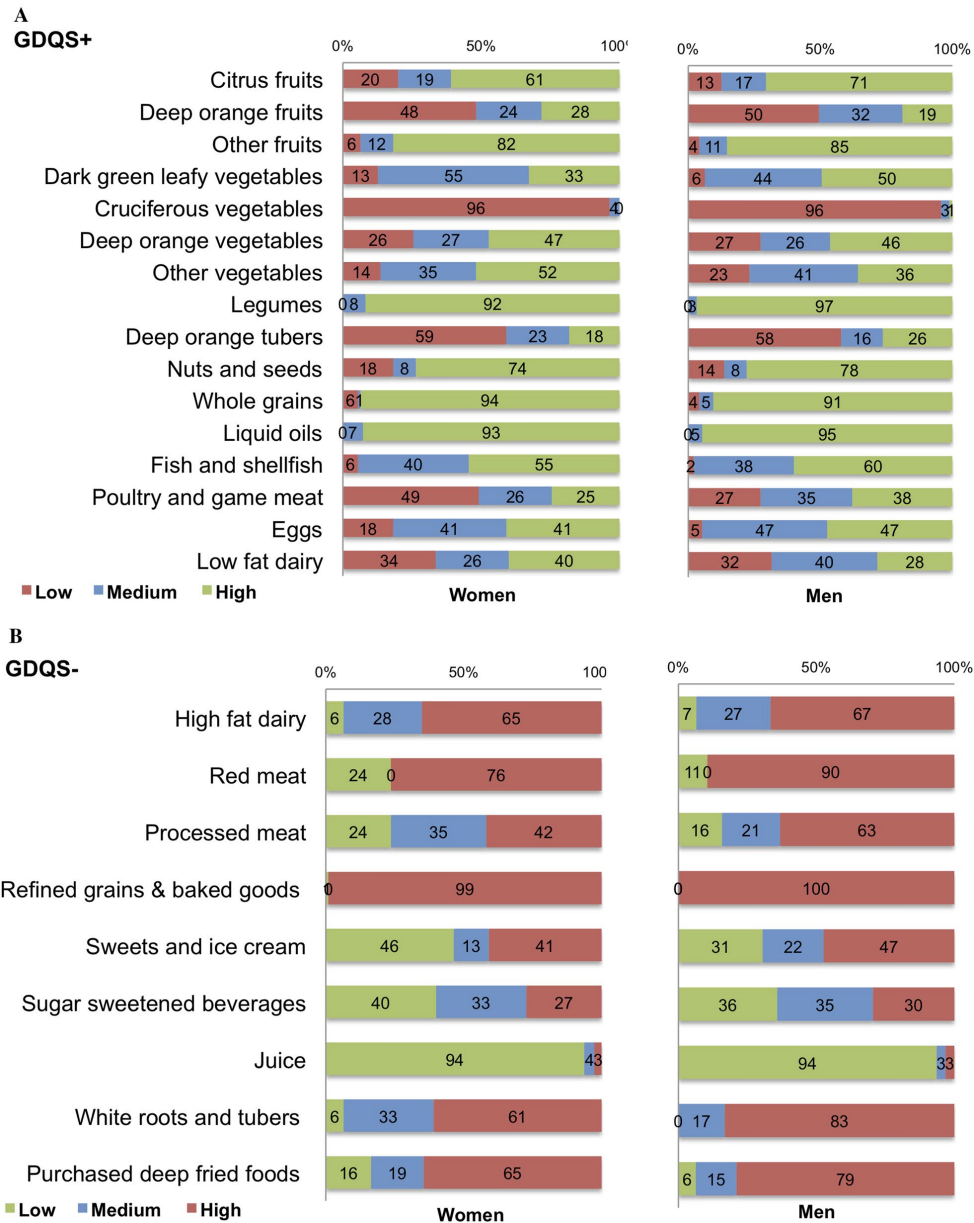
Percentage distribution of low, medium, and high intake of global diet quality score (GDQS) + (16 healthy food groups-that should be consumed in high amounts) and GDQS − (9 unhealthy food groups- that should be consumed in low amounts) in men and women. N = 205 baseline

Figure 2 shows the food groups that contributed the most to the differences in GDQS and its sub-metrics by sex.

### GDQS across seasons

The GDQS did not vary comparing dietary intakes during the rainy and the dry seasons in men (*p* = 0.57) and women (*p* = 0.49) (Table 4). Neither was there seasonal variation in the GDQS + among men (*p* = 0.48) or among women (*p* = 0.60), nor in the GDQS − among men (*p* = 0.65) or among women (*p* = 0.66).

**Table 4:** Season-based comparison of GDQS and Its healthy (GDQS +) and unhealthy (GDQS −) sub-metrics by sex

	Men			Women		
	Dry season	Rainy season	*p*-value*	Dry season	Rainy season	*p*-value*
	Mean (SD)			Mean (SD)		
GDQS	28.9 (3.9)	29.2 (3.5)	*p = 0.57*	28.5 (4.0)	29.0 (3.9)	*p* = 0.49
GDQS +	24.0 (4.2)	24.2 (3.9)	*p* = 0.48	22.7 (4.3)	23.0 (4.5)	*p* = 0.60
GDQS −	4.9 (1.8)	5.0 (1.9)	*p* = 0.65	5.7 (2.0)	5.9 (2.2)	*p* = 0.66

GDQS, global diet quality score

*P*-values less than 0.05 are considered statistically significant

^*^Wilcoxon signed-rank test

**Fig. 3:**
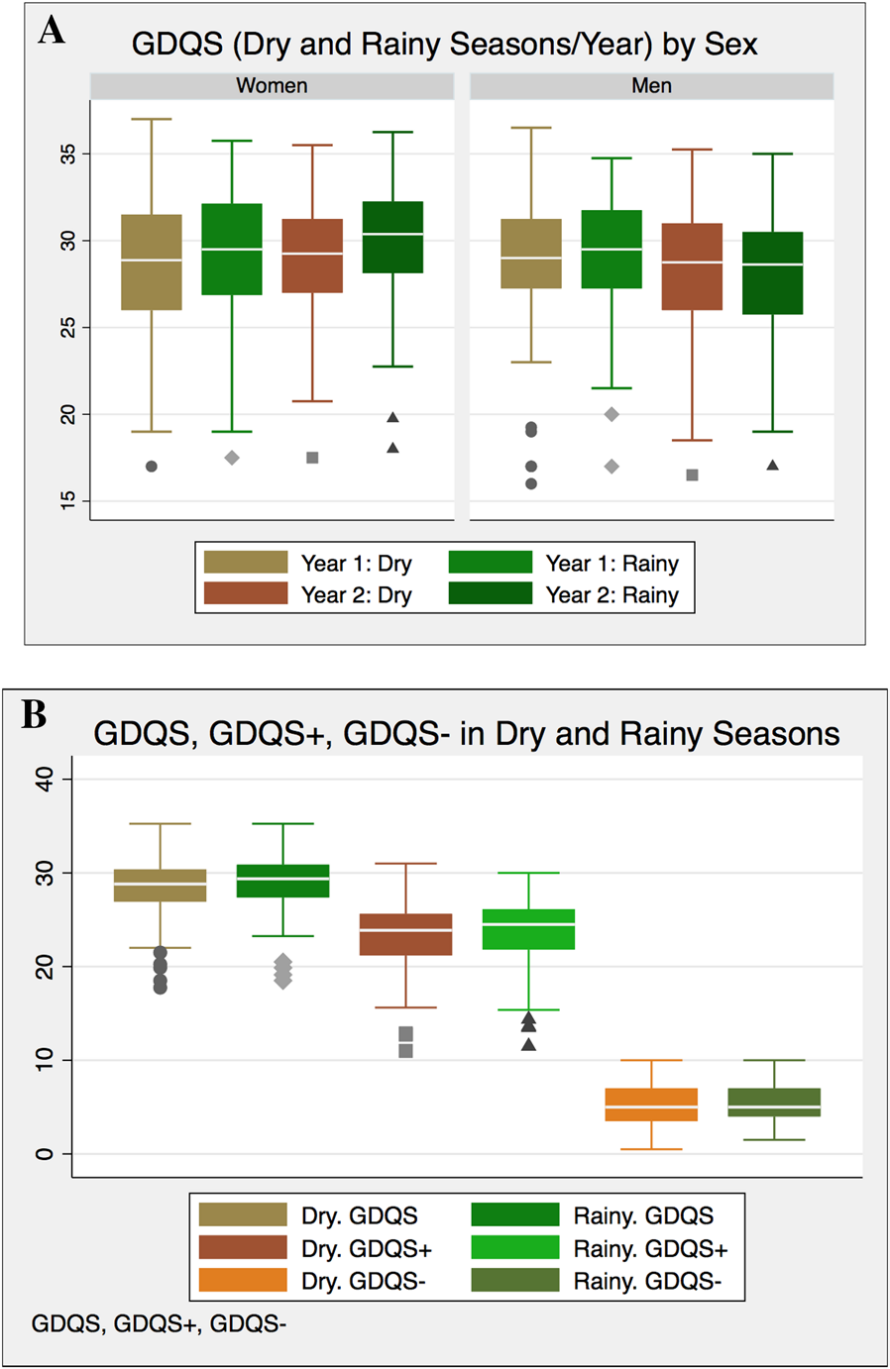
**A**. Changes in GDQS, global diet quality score (GDQS) across dry and rainy seasons in year 1 and year 2 for men and women. **B**. GDQS and its sub-metrics (GDQS + and GDQS −) across dry and rainy seasons

In years 1 and 2, GDQS and its sub-metrics remained consistent across all seasons for both sexes (Fig. 3).

### Factors associated with GDQS and its sub-matrices

Table 5 presents the factors associated with GDQS and its sub-metrics.

**Table 5:** Exploring factors associated with GDQS and sub-matrices: multivariable analysis and coefficient estimates in a baseline sample N = 205

		GDQS		GDQS +		GDQS −	
Socio-demographics		Coefficient (95% CI)	*p*-value*	Coefficient (95% CI)	*p*-value*	Coefficient (95% CI)	*p*-value*
Sex	Women	ref		ref		ref	
	Men	0.76 (− 0.60–2.11)	0.27	1.64 (0.11–3.03)	0.03	− 0.88 (− 1.53—− 0.24)	0.01
Age (years)	19–40	ref		ref		ref	
	41–50	0.44 (− 1.08–1.96)	0.57	− 0.71 (− 2.35–0.93)	0.4	1.14 (0.42–1.87)	0.002
	51–60	1.86 (0.17–3.56)	0.04	0.26 (− 1.56–2.09)	0.78	1.60 (0.80–2.41)	< 0.001
	60 +	0.41 (− 1.54–2.36)	0.68	− 2.19 (− 4.20–0.00)	0.05	2.51 (1.58–3.43)	< 0.001
Tribe	Yoruba	ref		ref		ref	
	Igbo	0.54 (− 1.09–2.17)	0.51	0.20 (− 1.54–1.95)	0.82	0.33 (− 0.42–1.09)	0.38
	Hausa/Fulani	0.13 (− 1.41–1.67)	0.87	1.78 (0.13–3.42)	0.04	− 1.64 (− 2.36− − 0.93)	< 0.001
	Other	− 0.39 (− 3.05–2.26)	0.77	− 0.87 (− 3.71–1.98)	0.55	0.48 (− 0.76–1.71)	0.45
Work	Unemployed	ref		ref		ref	
	Self-employed	0.79 (− 1.40–2.91)	0.49	1.00 (− 1.30–3.31)	0.39	− 0.24 (− 1.24–0.76)	0.63
	Skilled manual	0.92 (− 1.05–2.89)	0.36	1.42 (− 0.69–3.54)	0.19	− 0.50 (− 1.42–0.42)	0.28
	Professional/executive	2.48 (− 0.13–5.10)	0.06	2.24 (− 0.65–5.14)	0.13	− 0.24 (− 1.46–0.98)	0.69
Education	< 11 years of school	ref		ref		ref	
	11–12 years of school	1.32 (− 0.19–2.83)	0.09	0.57 (− 1.05–2.20)	0.49	0.63 (− 0.07–1.33)	0.08
	Post school/university	− 0.10 (− 1.94–1.74)	0.91	− 0.72 (− 2.70–1.26)	0.47	0.54 (− 0.33–1.41)	0.22
How frequently do you consume home− cooked meals?							
Dietary habits	Rarely/sometimes	ref		ref		ref	
	Most of the time	0.79 (− 0.88–2.47)	0.35	0.22 (− 1.57–2.02)	0.81	0.70 (− 0.10–1.47)	0.09
	All the time	2.04 (0.11–4.07)	0.04	1.73 (− 0.41–2.88)	0.11	0.33 (− 0.61–1.28)	0.49

CI, confidence interval; GDQS, global diet quality score; ref, reference category

*P*-values less than 0.05 are considered statistically significant

^*^Generalized Linear Model

Overall, there was a significant association between GDQS and the frequency of consumption of home-cooked meal, with a 2.04 difference in score (95% CI: 0.11–4.07) favoring those who consumed home-cooked meals all the time compared to those who rarely or sometimes consumed them. Men exhibited a 1.64 point higher score compared to women (95% CI: 0.11–3.03). Individuals from the Hausa/Fulani tribe showed a 1.78 point higher GDQS + score compared to those from the Yoruba tribe (95% CI: 0.13–3.42). For the unhealthy GDQS − sub-metric, men had a 0.88 point lower score compared to women (95% CI: − 1.53–− 0.24). Additional factors influencing the GDQS − included age and tribe. Individuals aged 60 years and older had a 2.51 point higher GDQS − score (95% CI: 1.58–3.43), while those from the Hausa/Fulani tribe had a 1.64 point lower score compared to those from the Yoruba tribe (95% CI: − 2.36–− 0.93).

## Discussion

In this study, most participants reported high intake of healthy foods and a low intake of quality foods as assessed by the GDQS. We found that the healthiness of food consumption was relatively consistent throughout the year and did not vary significantly between the rainy and dry seasons. There were no significant differences in overall GDQS across sex, age, education, work status, and ethnicity but participants who frequently ate home-cooked meals had better GDQS compared to those who did not. We also found differences in the GDQS sub-metrics (GDQS + , GDQS − ) by sex and age groups, particularly in the consumption of unhealthy foods (GDQS − ). There was a generational divide in food consumption with younger individuals tending to have higher intakes of unhealthy foods. Similarly, men, who were also more likely to eat away from home, had higher intakes of unhealthy foods.

Nigeria, like many LMIC, is undergoing a nutritional transition. The drivers of this dietary transition include urbanization, globalization, and an inclination towards emulating Western dietary practices [12, 23, 24]. We found differences in food consumption that are consistent with a wider "nutrition transition" in dietary intakes towards increased consumption of unhealthy food products, particularly among men and young adults [12, 23–25]. Despite the acknowledged speed with which this transition is occurring all over LMICs, our study shows that traditional Nigerian diets are still more prevalent and are included in the menu of the newly emerging fast-casual and take-away foods establishments in the country. This persistence my slow down the consumption of ultra-high processed foods and reduce their impact on health outcomes in Nigeria [26].

Previous studies have established associations between poor GDQS and unfavorable health outcomes, notably with type 2 diabetes [27], hypertension [28], and cardiovascular disease [29]. Although most of the participants in this study had a healthy diet with low intakes of poor-quality diets, there was very low to no consumption of certain important food groups regardless of sex or age group. This includes very low intake of cruciferous vegetables (broccoli, Brussels sprouts, cabbage, and cauliflower), fresh dairy products, and fresh dark green vegetables. The mean intake of cruciferous vegetables in our study was 1.5 g per day and more than 95% of our study participants did not report any intake of these vegetables. This contrasts with findings from the US and Europe, where 98% of participants in a US study reported consuming 24.0 (30.3)g (mean (SD)) of cruciferous vegetables per day [30]. Analysis in the European Prospective Investigation into Cancer and Nutrition (EPIC) cohort showed median daily intake of cruciferous vegetables of 6.16 g per day, varying from 0.37 g per day in Spain to 11.34 g per day in the UK [30]. Consumption of these vegetables is important because they are associated with reduced risks of several NCDs [31–36]. They are rich in nutrients such as carotenoids, vitamins (C, E, and K), folate, minerals, and fiber, and contain sulfur-containing glucosinolates, which have potential diseases prevention properties [33–37].

Our findings are similar to other studies in LMICs. Bromage *et al.* conducted secondary analysis of dietary intakes of rural men and women (age range = 15–49 years) in 10 African countries and found a mean (SD) GDQS of 26.3(3.4) in Nigeria [15]. This is similar to the GDQS we found in this study. The Nigerian diet has a relatively high mean GDQS, surpassing that observed in studies in Mexico (mean (SD) GDQS = 20.1(3.8)) [36], China (19.8(5.6)) [38], and Brazil (14.5(8.5)) [39]. This may be due to variations in consumption of legumes, fruits, vegetables, nuts, seeds, whole grains, high-fat dairy and processed meats, along with the relatively high prevalence of home-cooked meals in Nigeria.

Despite the perception that food availability, particularly in rural regions, is influenced by agricultural seasons and production cycles, we did not observe any significant seasonal variability in the GDQS and its sub-metrics in this study [10, 40]. This finding aligns with results from the Nigeria General Household Survey, which showed that the consumption patterns of food groups among both urban and rural households did not exhibit clear seasonal fluctuations, even for perishable items like vegetables and fruits [41].

Our study has several strengths. We had a large purposively selected sample and we conducted this study over a 2-year period encompassing 2 dry and rainy seasons with low dropout rate. By using a comprehensive FFQ specifically designed for the study population which included a listing of commonly consumed foods and integrating it with an FPB featuring standardized portion sizes, we enhanced the accuracy and precision of dietary intake reporting. Finally, in evaluating the overall healthiness of food consumption, we examined the GDQS and its sub metrics. Nevertheless, this study has several limitations. We recruited study participants solely from an urban area in southwest Nigeria, and the dietary intakes of urban residents in Nigeria may be significantly different from those of rural residents, though our findings were similar to that of a study conducted in rural Nigeria [15]. While we ensured diversity of tribes among our study participants, the diets of individuals from tribes not native to the city where we conducted our study may have been influenced by local dietary patterns and food availability. Nevertheless, we found differences in food consumption by tribe/ethnicity in this study. Our analysis of mixed dishes assumed uniform recipes [21], but variations may exist during preparation and ingredient choices [8], particularly considering the high prevalence of home-cooked meals in this study.

## Conclusions

Our research shows that while the overall healthiness of foods consumed by Nigerian adults remains relatively high across seasons, men and younger adults tend to consume more unhealthy foods and fewer home-cooked meals than women and older adults. This trend could negatively impact diet quality and contribute to the rising prevalence of non-communicable diseases (NCDs) in Nigeria. Notably, the low intake of certain food groups, such as cruciferous vegetables, has significant implications for public health, policy, and agricultural practices. Our findings suggest the need for targeted interventions that promote healthier dietary habits, especially among men and younger adults, and encourage the consumption of nutrient-rich foods. These interventions could be supported by policies that foster home-cooked meals and address socio-demographic factors influencing food choices. Ultimately, addressing these trends could mitigate NCDs and guide the integration of health, agriculture, and education policies.

## Data Availability

The datasets generated and/or analyzed during the current study are not publicly available due because additional analyses are being conducted on them but are available from the corresponding author on reasonable request.

## Abbreviations

CVD: Cardiovascular disease
FFQ: Food frequency questionnaire
FPB: Food picture book
GDQS: Global diet quality score
GLM: Generalized linear model
IRB: Institutional review board
LMIC: Low- and middle-income countries
NHREC: National health research ethics committee
REDCap: Research electronic data capture
